# Propositional and imagistic incongruity: a dual-route cognitive account of humor processing

**DOI:** 10.1007/s10339-026-01398-6

**Published:** 2026-09-25

**Authors:** Omid Khatin-Zadeh, Hassan Banaruee

**Affiliations:** 1 School of Foreign Languages, University of Electronic Science and Technology of China, Chengdu, China; 2 Department of Educational Psychology, University of Education Weingarten, Weingarten, Germany; 3 Institute for Innovative Teaching and Applied Teaching and Learning Research, University of Applied Sciences Ravensburg-Weingarten, Weingarten, Germany

**Keywords:** Humor, Dual-route account of humor, Imagistic incongruity, Propositional incongruity, Incongruity resolution

## Abstract

This paper develops a theoretical account of humor processing in terms of two interacting routes of cognitive processing. Building on the distinction between propositional and imagistic modes of language comprehension, it argues that humorous incongruity may arise through conceptual, perceptual, or cross-representational discrepancies. The propositional route involves relatively abstract and amodal representations, in which humor emerges from a mismatch between conceptual expectations and asserted or implied meanings. The imagistic route involves mental imagery, perceptual simulation, and sensorimotor representation, in which humor emerges from discrepancies between expected and activated perceptual or multimodal representations. In addition, the paper proposes that humorous effects may arise through between-route incongruity, where propositional and imagistic representations conflict, as in cases where verbal content and gesture, visual imagery, or embodied cues convey incompatible meanings. This dual-route account is not intended to replace existing theories of humor, but to specify a processing-level distinction that clarifies how incongruity is represented and resolved across verbal, visual, and multimodal forms of humor. By linking humor processing to conceptual processing, mental imagery, and embodied cognition, the account provides a framework for explaining why formally similar humorous expressions may differ in cognitive demands, perceptual salience, and affective impact. The paper concludes by outlining implications for future behavioral, cognitive, and multimodal research on humor processing.

## Introduction

Humor is a common aspect of human social life and plays a central role in social interaction (Martin 2003). Across disciplines, it is commonly treated as a complex and multidimensional phenomenon that engages cognitive, affective, perceptual, and interactional processes (Banaruee and Bieg 2026a, b; Martin 2003; Ruch et al. 2019). Yet despite its prevalence, humor has remained theoretically resistant to cognitive explanations. One reason for this difficulty lies in the fact that humorous phenomena cut across multiple levels of analysis, ranging from abstract conceptual reasoning to bodily sensation, perceptual experience, and real-time interpersonal coordination. Contemporary literature has not adequately examined humor processing in terms of imagistic and propositional modes; thus, we aim to address this gap in part.

In recent years, research in cognitive linguistics, embodied cognition, and interaction studies has increasingly converged on the view that humor is fundamentally grounded in embodied, affective, and multimodal processes (Gibbs et al. 2018). Embodied cognition represents a fundamental shift in how cognition and communication are conceptualized, challenging models that treat the mind as an abstract, amodal processor of symbolic information (Barsalou 1999, 2008; Shapiro 2019; Wilson 2002). Across diverse theoretical formulations, including perceptual symbol systems (Banaruee and Khatin-Zadeh 2026; Barsalou 2008), enactive and ecological approaches (Gibson 1977, 1986; Varela et al. 1991), and semiotic models of embodiment (Zlatev 2009), a set of broadly shared principles has emerged. These principles emphasize that cognition is grounded in sensorimotor systems, shaped by bodily and affective states, situated within physical and social environments, expressed through multiple modalities, and distributed across interacting individuals via coordination, synchrony, and co-regulation (Clark 1997; De Jaegher and Di Paolo 2007; Gallagher 2005, 2017; Niedenthal 2007; Richardson et al. 2007).

Applied to humor research, cognitive linguistic studies have demonstrated that humorous meaning often arises through embodied metaphor, sensorimotor simulation, and experiential knowledge, rather than through linguistic decoding alone (Attardo 2019; Gibbs et al. 2018; Samermit and Gibbs 2016; Xu et al. 2022). From this perspective, humor is not merely the outcome of abstract semantic computation but is closely tied to how individuals engage bodily with their physical and social environments. Gibbs et al. (2018), for instance, argue that humor, including irony and related phenomena, is rooted in bodily interaction and frequently emerges from benign violations of sensorimotor expectations. Further, bodily experience plays a constitutive role in how humorous expressions are conceptualized, produced, and interpreted in interpersonal contexts (Samermit and Gibbs 2016; Xu et al., 2022), revealing important limitations of approaches that treat humor as a purely linguistic or propositional phenomenon.

Empirical studies have shown that humor perception and amusement are modulated by bodily states such as posture, facial muscle activity, and affective resonance (Kaspar et al. 2016; Zheng and Wang 2023). Interactional studies likewise highlight the role of facial feedback, mirroring, and interpersonal synchrony in the emergence of humorous episodes (Attardo 2019). Amusement as a companion of funniness, in turn, has been found to feed back into ongoing cognitive processing through embodied and affective pathways, shaping attention, evaluation, and interpretation (Zheng and Wang 2023). The processing of incongruity and nonsense humor engages not only prefrontal regions associated with higher-order cognition but also sensorimotor systems involved in perception and action (Samson et al. 2009). Taken together, these converging strands of research align with embodied cognition approaches, which hold that cognition is grounded in sensorimotor activity, affective dynamics, and continuous interaction with the physical and social environment (Banaruee and Khatin-Zadeh 2026; Barsalou 2008; Shapiro 2019; Wilson 2002; Ziemke 2003; Zlatev 2009). According to the incongruity-resolution theory (Koestler 1964; La Fave et al. 1996), every humorous statement contains incongruent information and the perceived humor relies on two phases of recognition of that incongruent message and then its resolution as positive or negative humor. Along with these research findings and arguments, an important theoretical question remains insufficiently addressed: through which processing routes is humorous incongruity resolved? Although humor research increasingly acknowledges embodiment, multimodality, and interaction, most accounts continue to treat incongruity as a unitary mechanism. As a result, they offer limited explanations for why some humorous effects arise prior to explicit linguistic interpretation, why non-verbal or multimodal humor can succeed independently of verbal content, or why formally similar humorous expressions may be experienced differently across contexts and modalities.

The present paper addresses this gap by developing a dual-route account of humor processing grounded in insights from cognitive linguistics and incongruity theory (Koestler 1964; La Fave et al. 1996). Specifically, we build on the well-established distinction between the propositional and imagistic routes of language processing and extend this distinction to humor. Research in cognitive linguistics (e.g., Carston 2010; Ervas 2021; Indurkhya 2016; Khatin-Zadeh and Banaruee 2025; Khatin-Zadeh et al. 2026; Searle 1979) has long shown that linguistic meaning may be processed through at least two distinct routes: a propositional route, in which meaning is constructed through abstract, logic-based representations, and an imagistic route, in which meaning emerges through the activation and integration of mental imagery and sensorimotor representations. Drawing on this distinction, we argue that humorous incongruity likewise operates through two analytically distinguishable but interacting routes. On this account, humor may be produced through propositional incongruity, which is grounded in conceptual or amodal discrepancies between abstract representations, or through imagistic incongruity, which is grounded in perceptual or multimodal discrepancies involving imagery and sensorimotor experience. Importantly, humorous effects need not arise exclusively within a single route.

In addition to within-route incongruity (propositional-propositional or imagistic-imagistic), humor may also emerge from between-route incongruity, in which propositional and imagistic representations clash. This dual-mode perspective is not intended as a competing humor theory; rather, it offers a processing-level distinction that cuts across existing accounts and clarifies how humor operates across verbal, non-verbal, and multimodal interaction. Although the account is formulated with reference to humor processing broadly, it is important to clarify the scope of the present paper. Humor processing includes both production and comprehension: speakers or other communicators generate humorous effects by selecting, combining, or juxtaposing representations, whereas recipients interpret these effects by detecting and resolving incongruity. The present article is primarily concerned with the representational and resolution mechanisms that make such humorous effects possible. Thus, the focus is not on the full sequence of communicative planning involved in producing humor, nor on all affective and social outcomes of humor reception. Rather, the aim is to explain how humorous incongruity can be represented, detected, and resolved through propositional, imagistic, or combined propositional-imagistic routes. In this sense, the proposed framework is intended as a cognitive account of humor processing that can inform future research on both humor production and humor comprehension.

## Classical humor theories from an embodied perspective

Classical humor theories—superiority (Hobbes 2008), incongruity (Koestler 1964), and relief (Freud 1905)—have shaped humor scholarship for centuries and continue to influence contemporary research. These theories, however, were developed prior to current understandings of embodied cognition, multimodal communication, and interactional dynamics. Consequently, they have typically been interpreted in predominantly cognitive, emotional, or social terms, with limited attention to bodily and perceptual processes. From an embodied perspective, each of these theories can be re-examined to foreground dimensions that have remained under-theorized.

Superiority theory, for example, explains humor in terms of perceived hierarchies or feelings of relative advantage (Hobbes 2008). While often framed as a cognitive or moral judgment, this perspective can also be understood in embodied terms, involving bodily markers of dominance, affiliation, or threat. In interaction, humor associated with superiority is frequently accompanied by characteristic postures, facial expressions, and vocal qualities that signal relational stance. Interlocutors may thus respond to embodied cues of hierarchy or affiliation even before explicitly interpreting humorous content, suggesting that superiority-related humor is experienced through bodily and relational channels as well as through abstract evaluation.

Incongruity theory conceptualizes humor as arising from violated expectations and their resolution (Koestler 1964; La Fave et al. 1996). Traditionally treated as a cognitive mismatch, incongruity can also be understood in embodied terms. Expectations are formed not only with respect to verbal content but also with respect to timing, gesture, gaze, and interactional rhythm. Unexpected pauses, exaggerated movements, or playful shifts in posture may generate embodied forms of incongruity that are perceived prior to, or independently of, linguistic interpretation. Neurocognitive evidence showing multimodal integration during humor processing (Samson et al. 2009) is consistent with this broader, embodied understanding of incongruity.

Relief theory views humor as a release of psychological tension (Freud 1905). From an embodied standpoint, this release may be understood as the regulation of bodily arousal and affective states, involving changes in respiration, posture, facial musculature, and autonomic activity. Pro-social humor, in particular, may coincide with visible relaxation, shared laughter, and affective synchrony, producing cycles of tension and release that facilitate interpersonal alignment rather than merely intrapsychic discharge. Taken together, these reinterpretations do not displace classical humor theories but situate them within a broader embodied and interactional framework. By foregrounding bodily cues, affective resonance, and real-time coordination, an embodied reading reveals continuities between classical accounts and contemporary research, supporting the view that humor is not simply a cognitive judgment or verbal event but an embodied, socially organized phenomenon unfolding through interaction.

The remainder of the paper is organized as follows. Section  3 reviews the propositional and imagistic routes as two modes of language processing. Section  4 extends this distinction to humor, proposing corresponding propositional and imagistic routes of humor processing. Section  5 reconceptualizes these routes as amodal and multimodal mechanisms, respectively, and argues that humorous expressions may arise from both within-route and between-route incongruity.

## Propositional vs. imagistic route of language processing

The question of whether linguistic expressions are processed via a propositional or an imagistic route has been the subject of extensive debate in cognitive linguistics. The propositional route of language processing refers to a mechanism in which the meanings of the words constituting a linguistic expression are activated and combined without invoking a mental image of the situation described by that expression (Carston 2010, 2018; Carston and Wearing 2011; Indurkhya 2016; Wilson and Carston 2006, 2008; Wilson and Sperber 2004; for a review, see Wang 2025). This route is largely logic-based: propositional contents are activated and, when necessary, pragmatically adjusted to yield an interpretation that fits the contextual constraints (Carston 2010; Ervas 2021; Sperber and Wilson 1995, 2008; Wearing 2015). By contrast, the imagistic route of language processing involves the activation of mental images associated with words and, potentially, the integration of these images into coherent mental representations of the situation described (Carston 2010, 2018; Davidson 1978; Forceville 2020; Gibbs and Bogdonovich 1999; Indurkhya 2016; Wilson and Carston 2019). In other words, while meaning is encoded in linguistic forms, it may be decoded either through a propositional route, by activating and manipulating propositional content via logic-based processes, or through an imagistic route, by activating and integrating imagistic content via image-based processes.

The propositional route of language processing results in the construction of propositional meaning, whereas the imagistic route gives rise to imagistic meaning (Indurkhya 2016). For example, the sentence *the performance of the team is debated on social media* is primarily processed through the propositional route, as comprehension does not require the activation of mental images associated with either the team or social media. It should be noted that, during the processing of this literal sentence, mental imagery related to the team under discussion or to objects associated with social media may be activated. However, such activation does not make a significant contribution to sentence comprehension. In fact, the sentence can be readily understood without the activation of mental imagery, relying instead on the propositional route of language processing. By contrast, the sentence *the boxer is like a gorilla* is more likely to be processed through the imagistic route, since understanding the comparison typically involves activating a mental image of a gorilla and integrating it with the image of a boxer in order to infer the relevant similarities. Importantly, reliance on one processing route does not entail the complete exclusion of the other; a linguistic expression may be interpreted through a combination of propositional and imagistic processes (Carston 2010; Indurkhya 2016; Khatin-Zadeh and Banaruee 2025; Khatin-Zadeh et al. 2026). Although the distinction between propositional and imagistic routes applies to both literal and metaphorical language, it has been discussed predominantly in relation to metaphor. While some scholars, such as Grice (1975) and Searle (1979), adopted a propositional approach to metaphor processing and denied a substantive role for mental imagery (see also Black 1962; Glucksberg and Estes 2000; Vega Moreno 2007), others, most notably Davidson (1978), advanced an imagistic account, arguing that metaphor functions by prompting individuals to see one thing as another (for reviews, see Carston 2018; Khatin-Zadeh and Banaruee 2025).

In the following section, we build on incongruity theory of humor and the distinction between the propositional and imagistic routes of language processing to propose a parallel distinction between propositional and imagistic incongruity, which in turn underpins a distinction between propositional and imagistic routes of humor processing.

## Propositional vs. imagistic route of humor

As noted, incongruity theory (Koestler 1964; La Fave et al. 1996) holds that humor arises from a mismatch between what an individual expects and what actually occurs. Such incongruity can be realized in different ways, and a crucial issue concerns the route through which this discrepancy is processed. We propose that incongruity may be realized via the propositional route, the imagistic route, or a combination of the two routes of language processing. When the meaning of an expression is derived primarily through the propositional route, humor is produced propositionally; when meaning is derived primarily through the imagistic route, humor is produced imagistically. For instance, when the sentence *John is the cleverest student in our school* is used to describe a student who is in fact the laziest and lowest-performing in his class, the utterance is interpreted humorously. Because the meaning of this sentence is derived primarily through the propositional route of language processing, the resulting discrepancy is detected at the propositional level, and the humor is therefore produced via the propositional route. This interpretation arises because the key predicate *clever* denotes an abstract property that does not readily evoke mental imagery. We refer to this as *the propositional route of humor processing*. In this route, humor emerges from a propositional incongruity, that is, a logically grounded discrepancy between the propositional content of the expression and the actual properties of the entity described. In other words, the essence of the propositional route of humor lies in the mismatch between what is propositionally asserted and what is known or observed to be the case.

By contrast, when the sentence *Sam is the most powerful bodybuilder* is used to refer to an individual who is in fact very thin and physically weak, the utterance is primarily interpreted through the imagistic route of language processing, and an imagistic discrepancy is detected between what is said and what actually exists. Because the term *bodybuilder* denotes a concrete entity that is strongly associated with mental imagery, it readily activates a visual representation. When this image is juxtaposed with the image of *Sam* as a thin and weak individual, the resulting clash between the two images gives rise to humor. This incongruity is therefore predominantly imagistic rather than propositional, as it is not chiefly resolved through logical or inferential processes. We refer to this as *the imagistic route of humor processing*. In the imagistic route, humor arises from an imagistic incongruity between the image evoked by the linguistic expression and the image of the entity or situation to which the expression refers. In other words, the essence of the imagistic route of humor is the existence of a sharp discrepancy between the imagistic meaning or imagistic content of an expression and the image of the object described by that expression. In imagistic language processing, words function as cues for mental images that are normally expected to align with their referents; when this expectation is violated through an unexpected discrepancy, imagistic humor is produced. To clarify the explanatory function of the proposed distinction, consider four cases in which humorous incongruity is produced through different representational routes.

First, propositional incongruity can be illustrated by the utterance “Our committee is famous for making quick decisions,” used in a context where the committee has postponed a simple decision for several months. The humorous effect does not depend primarily on a vivid mental image of the committee. Rather, it depends on the listener’s access to two incompatible propositional contents: the assertion that the committee is quick and the contextual knowledge that the committee is slow and indecisive. The activated representation is therefore conceptual and evaluative. The incongruity occurs between the proposition expressed by the utterance and the proposition made available by the context. This case supports the propositional route because the humorous effect is produced by a contradiction between abstract meanings, not by a conflict between perceptual images.

Second, imagistic incongruity can be illustrated by the utterance “He entered the room like a lion,” used to describe a person who walks in hesitantly, avoids eye contact, and immediately hides behind others. In this case, the expression activates an imagistic representation of a lion, including strength, confidence, bodily expansion, and dominance. This image is then compared with the perceptually available image of the person’s timid bodily behavior. The incongruity occurs between the image evoked by the linguistic expression and the image of the actual referent. Although the utterance also has propositional content, the humor depends strongly on the contrast between two embodied images: the expected image of a powerful entrance and the perceived image of a fearful entrance. This case supports the imagistic route because the humorous effect is produced through a perceptual and sensorimotor discrepancy.

Third, mixed propositional–imagistic incongruity can be illustrated by the utterance “Our economy is standing tall again,” used while the speaker refers to a graph showing a sharp economic decline. The verbal expression activates both a propositional meaning and an imagistic schema. Propositionally, it implies that the economy is improving or stable. Imagistically, the phrase “standing tall” activates an embodied verticality schema associated with upwardness, strength, and stability. The graph, however, presents a downward trajectory. The incongruity is therefore mixed. At one level, there is a propositional contradiction between the claim of improvement and the evidence of decline. At another level, there is an imagistic contradiction between the upward bodily schema evoked by “standing tall” and the downward visual pattern displayed in the graph. This case supports the mixed route because humor emerges from the simultaneous conflict between conceptual meaning and perceptual representation.

Fourth, multimodal incongruity can be illustrated by a speaker who says “Everything is going up” while making a clear downward hand gesture. The verbal channel activates an upward representation, both propositionally, through the claim of increase, and imagistically, through the spatial schema of upward movement. The gesture activates an incompatible visual and motor representation of downward movement. The incongruity occurs across modalities, since the verbal mode and the gestural mode convey conflicting directional meanings. The listener detects the contradiction not only by interpreting the sentence, but also by integrating the sentence with the speaker’s bodily action. This case supports the multimodal route because the humorous effect is produced through cross-modal conflict between speech and gesture.

The same route distinction also clarifies how incongruity is resolved. In propositional humor, resolution occurs mainly through pragmatic reinterpretation, schema adjustment, or bridging inference. The recipient recognizes that the literal or asserted meaning is incompatible with contextual information and then reconstructs a non-literal, ironic, exaggerated, or otherwise humorous interpretation. In imagistic humor, resolution depends more strongly on the restructuring or reinterpretation of perceptual and sensorimotor representations. The recipient detects a conflict between an expected image and an activated or perceived image, and the humorous effect emerges when this perceptual discrepancy is integrated into a coherent interpretation. In between-route humor, resolution requires the integration of conflicting information across representational channels, such as verbal content, visual display, gesture, posture, or bodily action. Humor therefore arises not from unresolved interference, but from the recipient’s ability to recognize the conflict as meaningful, intentional, or contextually interpretable. In this sense, resolution is not a single mechanism operating uniformly across all humorous material, but a route-sensitive process that may involve conceptual inference, imagistic restructuring, or multimodal integration.

These examples show that the proposed distinction is not limited to abstract classification. It identifies where the relevant incongruity is represented and processed. In propositional humor, the critical discrepancy is located between conceptual contents. In imagistic humor, it is located between perceptual or sensorimotor representations. In mixed cases, conceptual and imagistic discrepancies operate together. In multimodal cases, incongruity is distributed across semiotic channels, such as speech, gesture, image, or visual display. The dual-route account therefore provides a processing-level explanation of how different kinds of humorous material generate incongruity.

In the following section, we discuss the propositional route of humor processing as a relatively amodal mechanism and the imagistic route as a relatively multimodal mechanism. This formulation does not imply that propositional humor is entirely detached from embodied experience. Rather, it indicates that propositional humor depends more centrally on conceptual and inferential processing, whereas imagistic humor depends more centrally on perceptual, sensorimotor, and multimodal representations.

## Amodal vs. multimodal humor

As noted above, in the propositional route of language processing, the propositional component of meaning is activated. This route does not involve mental imagery or sensorimotor activation (e.g., Carston 2010, 2018; Carston and Wearing 2011; Ervas 2021; Indurkhya 2016; Wilson and Carston 2006, 2008; Wilson and Sperber 2004) and can therefore be characterized as an amodal route of language processing. By contrast, in the imagistic route of language processing, the imagistic component of meaning is activated, and interpretation involves mental imagery and sensorimotor activation. Importantly, mental imagery is not limited to visual properties but may also include auditory and kinetic properties (Carston 2018). The imagistic route is thus a multimodal route of language processing, engaging perceptual and sensorimotor systems. On this basis, the propositional route of humor processing can likewise be regarded as amodal. This route involves a propositional, or amodal, incongruity that is constructed by the speaker and recognized by the listener through logical and conceptual processes. While propositional humor processing is primarily handled by higher-level conceptual mechanisms, the imagistic route of humor processing is more closely associated with perceptual processes and perceptual systems. Accordingly, propositional humor arises from a conceptual or amodal discrepancy, whereas imagistic humor arises from a perceptual or multimodal discrepancy. Put differently, propositional humor is produced by what is conceptually unexpected, whereas imagistic humor is produced by what is perceptually unexpected.

It should be emphasized, however, that the presence of a concrete word does not by itself require imagistic processing. Many concrete expressions can be interpreted rapidly through abstract semantic features without sustained mental imagery or sensorimotor simulation. For example, a listener may understand the contrast between weak and strong by accessing propositional labels rather than by constructing a detailed bodily image. The imagistic route is therefore not assumed to be activated whenever a concrete noun, physical property, or bodily description appears in a humorous expression. Rather, imagistic processing is most likely to be central when the humorous effect depends on perceptual salience, bodily detail, gesture, spatial layout, visual contrast, or mental simulation. In such cases, the incongruity cannot be fully explained by abstract semantic opposition alone because the listener must compare, transform, or integrate perceptual and sensorimotor representations. This boundary condition helps distinguish genuine imagistic humor from cases in which concrete language merely supplies semantic content for propositional incongruity.

The distinction between propositional/conceptual and imagistic/perceptual humor is important because these can be understood as operating at two different levels of humor processing. Propositional or conceptual humor, as the higher level of humor, is produced through higher-level conceptual processes that involve amodal cognitive systems. The essence of this level of humor processing lies in conceptual unexpectedness, or conceptual incongruity, which is computed through abstract, high-level or abstract cognitive mechanisms. By contrast, imagistic or perceptual humor, as a low-level perceptual/sensorimotor processing level, is produced through perceptual and sensorimotor processes involving perceptual systems. The essence of this level of humor processing lies in perceptual unexpectedness, or perceptual incongruity, which is computed through low-level perceptual processes and multimodal systems. For example, when the sentence *this is a truly democratic country* is used to refer to a country that is in fact ruled by a dictator, the utterance is interpreted humorously. This humor is propositional in nature because it arises from a conceptual incongruity—indeed, a contradiction—between the abstract concept of *democracy* and the existence of a *dictator* as the country’s ruler. As Borghi et al. (2017) note, abstract concepts are unbounded and lack clearly perceivable properties. Both *democracy* and *dictator* denote abstract qualities that do not have salient perceptual features. In this case, humor arises from the fact that the abstract property of democracy is fundamentally incompatible with the concept of a dictator and can only be attributed to it for humorous effect. The listener recognizes the humorous nature of the expression by resolving the conceptual incongruity between the concepts of *democracy* and *dictator*.

While propositional/conceptual humor operates through an amodal route, imagistic/perceptual humor operates through a multimodal route. For example, when the sentence *our economy has an upward trend* is accompanied by a downward gesture indicating that the economy is actually performing poorly, humor arises through the imagistic/perceptual route. In this case, there is an imagistic incongruity between the verbal message and the gestural depiction. This constitutes multimodal humor because the information conveyed through one mode (verbal) is incongruent with the information conveyed through another mode (visual). Notably, good economic performance is metaphorically described in terms of upward movement (e.g., *the economy is rising*), which is processed through the imagistic mode of language processing. However, when this verbal expression co-occurs with a downward gesture, a cross-modal incongruity emerges. This highlights a key difference between propositional/conceptual and imagistic/perceptual humor: propositional/conceptual humor is amodal and relies on abstract cognitive processes, whereas imagistic/perceptual humor can be multimodal and engages perceptual systems across different modalities.

In the following section, we discuss the propositional route of humor processing as the result of an incongruity between two symbolically-represented elements and the imagistic route of humor processing as the result of an incongruity between two inherently continuous images that cannot be integrated into a coherent continuous image.

## Discrete vs. continuous incongruity

As mentioned, in propositional or conceptual humor, the humorous effect arises from a conceptual incongruity between two or more elements. This type of incongruity does not lend itself to a direct or clear visual representation; rather, it is articulated and processed through discrete linguistic symbols. In other words, conceptual incongruity is identified by activating and juxtaposing elements of meaning that are encoded linguistically. For example, when the sentence *John was the golden boy in the history of the Olympics* is used to describe an athlete who, in reality, performed very poorly in Olympic competitions, a clear conceptual incongruity emerges. The humor stems from the contradiction between what the term *John* refers to in the given context and the evaluative meaning conventionally associated with the expression *golden boy*. In this case, *John* and *golden boy* function as two distinct conceptual entities, each represented by discrete linguistic symbols. More generally, the context described by such a sentence is composed of a set of discrete elements that are combined and interpreted through language. Since language itself is a symbolic system made up of discrete units, the propositional humor produced in this example results from a logical mismatch between discrete meanings represented by arbitrary linguistic signs. In contrast, imagistic or perceptual humor involves a clearly perceivable incongruity between elements within a continuous mental image. Like language, mental imagery can be understood as a system of symbols that stand for elements in a described situation. However, imagistic symbols differ fundamentally from linguistic symbols in that they are embedded within continuous perceptual representations rather than being segmented into discrete units. As a result, imagistic humor emerges from an incongruity between elements that coexist within a unified and continuous image, rather than from a contradiction between abstract, linguistically encoded concepts.

Another important distinction concerns the relationship between symbols and their referents. Linguistic symbols have an arbitrary relationship to what they denote, and propositional humor arises from an incongruity between the meanings associated with these arbitrary symbols. Both the symbols themselves and their referents are treated as discrete entities. By contrast, imagistic symbols have a non-arbitrary relationship with their referents, as they typically resemble or directly mirror perceptual features of the objects or situations they represent. Consequently, both imagistic symbols and their referents possess a continuous nature. Therefore, the discrete structure of propositional humor and the continuous structure of imagistic humor can be regarded as a key difference between these two types of humor, shaping how incongruity is represented, processed, and ultimately experienced.

The distinction between discrete and continuous incongruity should therefore be understood as a processing continuum rather than as a rigid dichotomy. Discrete and continuous incongruity represent analytic poles that help identify the dominant representational format involved in a humorous effect, but they are not mutually exclusive categories. Many humorous cases involve hybrid configurations. Political cartoons, visual puns, and multimodal memes, for example, often use continuous visual images to convey discrete symbolic or propositional meanings. Conversely, discrete verbal expressions, especially metaphors and vivid descriptions, may rapidly trigger continuous mental simulations. The proposed distinction should therefore not be interpreted as implying that propositional humor is purely discrete or that imagistic humor is purely continuous. Rather, the point is that humorous incongruity may vary in the extent to which it depends on discrete conceptual-symbolic representations, continuous perceptual-imagistic representations, or their interaction.

## Within-route vs. between-route incongruity

As noted, propositional humor arises from an amodal incongruity between two or more conceptual elements within the described situation. This type of incongruity is classified as a *within-route* incongruity because all of the incongruent elements operate at the same representational level: they are conceptual in nature and encoded through linguistic symbols. In such cases, humor emerges from a mismatch between meanings that are accessed and processed within the conceptual–linguistic system, without reliance on perceptual or sensory information. Similarly, imagistic humor arises from a modal or embodied incongruity between perceivable elements. This, too, constitutes a within-route incongruity, since the incongruent elements are entirely perceptual and are represented in the form of mental images or directly perceivable stimuli. In imagistic humor, the humorous effect is generated through a conflict within the perceptual domain itself, such as visual or spatial inconsistencies, rather than through abstract conceptual contradiction. Thus, regardless of whether the incongruent elements are exclusively amodal (conceptual) or exclusively modal (imagistic), the resulting humor can be described as within-route humor. Accordingly, within-route humor can be further divided into within-route amodal humor (propositional humor) and within-route modal/multimodal humor (imagistic humor).

However, not all humorous situations are confined to a single route of exclusively amodal or exclusively modal/multimodal. In some cases, humor is produced through a *between-route* incongruity, in which elements from different representational systems conflict with one another. Specifically, when a conceptual element represented symbolically through language is incongruent with a clearly perceivable element represented imagistically, a between-route incongruity occurs. This type of incongruity involves interaction between conceptual–linguistic processing and perceptual processing. For example, consider a speaker criticizing poor economic conditions who utters the sentence *our economy is doing very well* while simultaneously using a downward hand gesture to indicate decline. In this scenario, a between-route incongruity arises because the conceptual meaning conveyed by the linguistic expression *doing very well* directly contradicts the imagistic meaning conveyed by the downward gesture, which is visually perceivable. What is expressed and interpreted propositionally through language is therefore incongruent with what is expressed and interpreted imagistically through gesture. The humor emerges precisely from the listener’s ability to integrate information across these two routes and detect the contradiction between them.

Between-route incongruity may also vary as a function of temporal dynamics. In natural interaction, gesture, posture, facial expression, and other visual cues do not always occur simultaneously with the relevant verbal expression. They may precede, accompany, or follow the linguistic material. Evidence from gesture-speech production indicates that the temporal relation between hand gestures and their lexical affiliates is systematically variable, with gesture-speech asynchrony predicted by word familiarity (Morrel-Samuels and Krauss 1992). When a gesture precedes speech, it may establish an imagistic expectation that is later confirmed or violated by the verbal proposition. When gesture and speech occur simultaneously, the recipient may integrate the two channels in parallel and detect incongruity as a conflict between concurrent representational streams. More generally, gesture is closely related to thought and speech rather than being a merely decorative accompaniment to verbal expression (McNeill 1992). This view is also consistent with evidence that iconic and metaphoric gestures vary across different types of motion-based, space-based, object-based, and event-based statements, suggesting that gesture can reflect distinctions in the conceptual and imagistic structure of the represented content (Khatin-Zadeh et al. 2022). In natural interaction, gesture, posture, facial expression, and other visual cues do not always occur simultaneously with the relevant verbal expression. ERP evidence indicates that gestures and speech are integrated during comprehension, with mismatching gestures eliciting semantic integration effects relative to matching gestures (Kelly et al. 2004). Related studies further show that semantic information from speech and gesture is integrated online and that iconic gestures can contribute to the disambiguation of spoken meaning, suggesting that gesture is not merely an external accompaniment to speech but participates in real-time semantic interpretation (Holle and Gunter 2007; Özyürek et al. 2007). When a visual or gestural cue follows the verbal expression, it may retrospectively reframe the utterance and create a delayed humorous interpretation. Thus, between-route humor should not be understood as a purely serial process in which verbal meaning is first computed and visual or bodily information is added later. Rather, it can involve temporally distributed and partially parallel integration across linguistic, visual, and sensorimotor channels. The salience of the humorous incongruity may therefore depend on the temporal relation between modalities, including whether imagistic information appears before, during, or after propositional information.

The humor produced through such between-route, or amodal–multimodal, incongruity can be regarded as following an amodal–multimodal route of humor processing. On this basis, three distinct routes of humor processing can be identified: propositional, imagistic, and propositional–imagistic. The first route constitutes an amodal pathway for humor processing, relying exclusively on conceptual systems. The second route constitutes a multimodal pathway, engaging perceptual systems. The third route integrates both amodal and multimodal processing, simultaneously engaging conceptual and perceptual systems. Together, these three routes represent distinct cognitive mechanisms through which incongruity is constructed, detected, and experienced as humorous.

## Conclusion

This paper proposed a dual-route cognitive account of humor processing. The central claim is that humorous incongruity can be represented and processed through two distinguishable but interacting routes. The propositional route is primarily associated with conceptual and inferential processing. In this route, humor emerges when the meaning asserted or implied by an expression conflicts with contextual knowledge, background assumptions, or another conceptual representation. The imagistic route is primarily associated with mental imagery, perceptual simulation, and sensorimotor representation. In this route, humor emerges when an evoked image, bodily schema, or perceptual expectation conflicts with another image, perceived situation, or multimodal cue.

The proposed distinction also allows for mixed and multimodal cases. Some humorous expressions involve both propositional and imagistic incongruity, as when an utterance conveys an abstract evaluative meaning while also activating a spatial, bodily, or visual schema that conflicts with the surrounding context. Other cases involve incongruity across semiotic channels, such as speech and gesture, verbal description and image, or linguistic content and visual display. In such cases, humor depends on the integration of information across representational systems. The dual-route account therefore does not treat humorous incongruity as a single undifferentiated mechanism. Rather, it specifies where the relevant incongruity is located and how it may be processed at conceptual, imagistic, or cross-representational levels.

This account contributes to the study of cognitive processing by linking humor processing to broader mechanisms of conceptual representation, mental imagery, sensorimotor simulation, and multimodal integration. It also clarifies why formally similar humorous expressions may differ in processing demands. Some cases require mainly the detection of conceptual contradiction, whereas others require the construction, comparison, or integration of perceptual and embodied representations. The examples discussed in the paper show that the proposed distinction has descriptive value for verbal humor, imagistic humor, mixed verbal-imagistic humor, and multimodal humor involving gesture or visual display.

The account is not intended to replace existing humor theories, including incongruity-resolution approaches. Instead, it complements them by adding a processing-level distinction. Incongruity remains central to humor, but the present account argues that incongruity may be represented in different cognitive formats. It may be propositional, imagistic, or distributed across propositional and imagistic systems. This distinction helps explain why some humorous effects are resolved through abstract conceptual interpretation, whereas others depend more directly on perceptual salience, bodily simulation, or multimodal coordination.

Several issues remain open for future research. The present paper is theoretical and does not test whether one route is more effective, faster, more cognitively demanding, or more affectively powerful than another. Future behavioral, neurocognitive, eye-tracking, corpus-based, and multimodal studies could examine how propositional and imagistic routes operate during real-time humor comprehension and production. More specifically, propositional incongruity could be examined through semantic-distance paradigms in which the conceptual distance between a setup and a punchline, or between an asserted meaning and contextual information, is quantified using human ratings or computational semantic models. For example, semantic-distance tools such as SemDis have been developed to quantify the semantic relatedness or distance between verbal responses and have shown substantial correspondence with human ratings of creativity and novelty (Beaty and Johnson 2021). More recent transformer-based approaches also indicate that automated assessment of divergent thinking can be extended beyond traditional semantic-distance models. For example, TransDis uses transformer-based language models to assess divergent thinking in Chinese and provides a computational framework for estimating originality and flexibility in creative idea generation (Yang et al. 2024). Such approaches could be adapted in future humor research to quantify propositional incongruity, semantic remoteness, or flexibility of reinterpretation across different languages. Relatedly, forward-flow methods quantify the semantic evolution of thought over time by estimating how far current thoughts move away from previous thoughts in a free-association sequence, and such measures have been shown to predict creative performance across laboratory and real-world contexts (Gray et al. 2019). In a similar vein, divergent-association paradigms ask participants to generate unrelated words and then use computational algorithms to estimate the average semantic distance among those words, with greater semantic distance predicting performance on established creativity measures (Olson et al. 2021). Individual-difference approaches could also examine whether verbal fluency and broad retrieval ability predict the generation or resolution of propositional humor. Because divergent thinking is partly supported by broad retrieval ability and verbal fluency (Silvia et al. 2013), future research could test whether individuals with stronger retrieval abilities are better able to access remote semantic associations, reinterpret incongruent propositions, or generate alternative humorous resolutions. Such paradigms could be adapted to humor research by examining whether individuals who generate or resolve more propositionally incongruent humorous material also show greater capacity to move between semantically distant concepts. Recent work on associative creativity further suggests that free and controlled associations may contribute jointly to creative cognition rather than operating as mutually exclusive processes (Liu et al. 2025). Related evidence also suggests that creative performance may depend on the interaction between controlled shifting, idea referencing, working memory capacity, and mind-wandering profiles (Yang et al. 2026). For humor research, this suggests that future studies should examine not only whether individuals can move toward remote semantic associations, but also how they strategically shift between interpretations and use prior ideas as references when constructing or resolving humorous incongruity. This distinction is relevant for future humor research because propositional and imagistic incongruity resolution may involve both spontaneous associative shifts and more controlled reinterpretive processes, depending on the type of humorous material and the cognitive demands of the task.

Applied to humor research, this approach could help examine whether the resolution of propositional incongruity involves a measurable shift from an initially expected semantic frame toward a more remote, playful, or nonliteral interpretation. This interpretation is also supported by reviews of metaphorical embodiment showing that metaphor comprehension is frequently grounded in perceptual, motor, affective, and bodily experience rather than being reducible to amodal semantic computation alone (Khatin-Zadeh et al. 2023). Although such tools were developed primarily for creativity assessment, they could be adapted to estimate the degree of conceptual remoteness or mismatch involved in propositional humor. Such designs would allow researchers to test whether greater propositional mismatch predicts perceived funniness, processing time, or resolution difficulty. Imagistic incongruity could be examined through visual-spatial mismatch tasks, mental imagery tasks, mental rotation paradigms, or motor-interference designs in which participants process humorous material that depends on bodily, spatial, or perceptual simulation. If imagistic humor depends more strongly on sensorimotor representation, then disrupting relevant perceptual or motor processes should affect its comprehension more than it affects primarily propositional humor. Between-route incongruity could be studied through multimodal paradigms that manipulate the relation between speech, gesture, image, and visual display. Eye-tracking, response-time measures, event-related potentials, and video-based interaction analysis could be used to examine when recipients detect conflicts across channels and how temporal alignment between verbal and imagistic information influences humor resolution. These paradigms would make it possible to operationalize the proposed routes and test whether propositional, imagistic, and between-route humor differ in cognitive load, temporal profile, and affective response. Such work could also investigate whether different humor genres, communicative contexts, or individual differences are associated with stronger reliance on one route or with more complex interaction between routes. By distinguishing conceptual, imagistic, and cross-representational incongruity, the dual-route account provides a framework for future empirical research on humor as a cognitive, embodied, and multimodal phenomenon.

## Data Availability

No datasets were generated or analysed during the current study.
